# Asymmetric Dimethylarginine does not Predict Early Access Events in Hemodialysis Patients with Brachiocephalic Fistula Access

**DOI:** 10.16966/2380-5498.141

**Published:** 2017-04-06

**Authors:** Mary Hammes, Rita McGill, Promila Dhar, Rama S Madhurapantula

**Affiliations:** 1 Department of Medicine, Section of Nephrology, University of Chicago, Chicago, IL; 2 Department of Biomedical Engineering, Illinois Institute of Technology, Chicago, IL; 3 Department of Biological and Chemical Sciences, Illinois Institute of Technology, Chicago, IL

**Keywords:** Arteriovenous fistula, Endothelium, Hemodialysis, Nitric Oxide, Stenosis, Thrombosis

## Abstract

**Background:**

Vascular access for hemodialysis is best provided by an arteriovenous fistula (AVF). AVF fail primarily because of neointimal hyperplasia. Asymmetric dimethlyarginine (ADMA) is a naturally occurring analogue of L-arginine, which is elevated in renal failure and impairs endothelial cell function. ADMA inhibits nitric oxide synthetase, leading to impaired nitric oxide production and contributing to the development of neointimal hyperplasia. ADMA was measured at the time of AVF placement to evaluate associations with access failure.

**Methods:**

ADMA was measured at the time of brachiocephalic access placement. Patients were followed for up to 12 months with end-points of access thrombosis or venous stenosis.

**Results:**

Sixty patients with primary brachiocephalic fistulas were included in the study cohort. The median value for ADMA drawn at the time of AVF creation was 3.1 µmol/L. ADMA was not significantly associated with early thrombosis or venous stenosis events (*P*>0.05).

**Conclusion:**

Preoperative ADMA levels, as a surrogate for endothelial cell dysfunction and predictor of adverse access event (thrombosis or stenosis), were not associated with subsequent access events Future studies that identify markers of endothelial cell dysfunction are warranted.

## Introduction

Patients with end-stage renal failure on hemodialysis require a vascular access which is best provided by arteriovenous fistulas (AVFs). AVF are preferred over other techniques for vascular access as they are less likely to need repeated interventions to maintain patency and function [1]. An AVF is created by a connection between artery and vein. The process of vascular maturation occurs over several weeks or months, which enables an AVF to become strong enough to withstand the powerful flows and pressures required for hemodialysis.

The anatomic location and configuration of an AVF is important to maturation and is particularly important in diabetics who are at higher risk for stenosis [2]. Lower arm (radiocephalic location) has a higher failure rate due to venous stenosis at the anastomosis. Therefore, AVFs are increasingly being placed in the upper arm (brachiocephalic location), especially in patients with diabetes [2]. The most common location for stenosis in the brachio-cephalic fistula (BCF) is in the cephalic arch [3], which occurs less commonly in diabetics [4,5].

The process of maturation of an AVF is complex and AVFs often fail prior to use. Intensive multi-disciplinary efforts have been expended to understand the biology and hemodynamics of arterial and venous dilation that result in AVF maturation [6]. After an AVF is created there is turbulent blood flow causing increased wall shear stress (WSS), resulting in production of endothelial nitric oxide synthetase, which converts arginine to nitric oxide (NO) [7]. NO dilates both the arterial and venous vessels to restore WSS to baseline and inhibits neointimal hyperplasia (NH) [8–10]. This process is tightly regulated and necessary for physiological function. NO generation is dependent upon a healthy endothelium. In end-stage renal disease (ESRD) there is often dysfunctional endothelial response to NO resulting in defective dilation and excessive intimal hyperplasia, which in turn lead to poor maturation, primary thrombosis, and AVF failure [11,12]. It would be prudent to explore and establish biomarkers which could be used as a predictive tool for AVF success and adverse events. However, we have no such biomarkers available in clinical use to predict events.

Asymmetric dimethylarginine (ADMA) is an intracellular amino acid analogue formed during post-translational methylation of arginine by methyltransferases. Under physiological conditions, ADMA is present in plasma, urine, and cells of many tissues. The concentration of ADMA in healthy individuals varies from 0.3–1.2 µmol/L [13,14]. ADMA concentrations have been shown to be higher in women and to increase with age [14]. A majority of ADMA is removed by the kidneys after metabolic conversion to citrulline and dimethylamine catalyzed by dimethyllarginine dimethylaminohydrolase (DDAH).

ADMA inhibits nitric oxide synthetase, leading to impair NO production and contributing to impairment of endothelial function, which in turn contributes to decreased vessel elasticity [15,16]. ADMA concentration is markedly increased in chronic kidney disease (CKD) and ESRD [17,18]. Plasma ADMA has been shown to predict restenosis of AVF six months after percutaneous interventions [19]. Recently, the Hemodialysis Fistula Maturation trial showed that pre-operative flow mediated dilation and nitroglycerin-mediated dilation were associated positively with postoperative AVF blood flow at 6 weeks [20]. Carotid-radial pulse wave velocity, which assesses the stiffness of the arterial circulation, did not have an association with 6 week AVF blood flow [20]. However, ADMA, which may have been a factor contributing to vascular stiffness and poor AVF maturation, was not measured in this trial.

**Figure 1:**
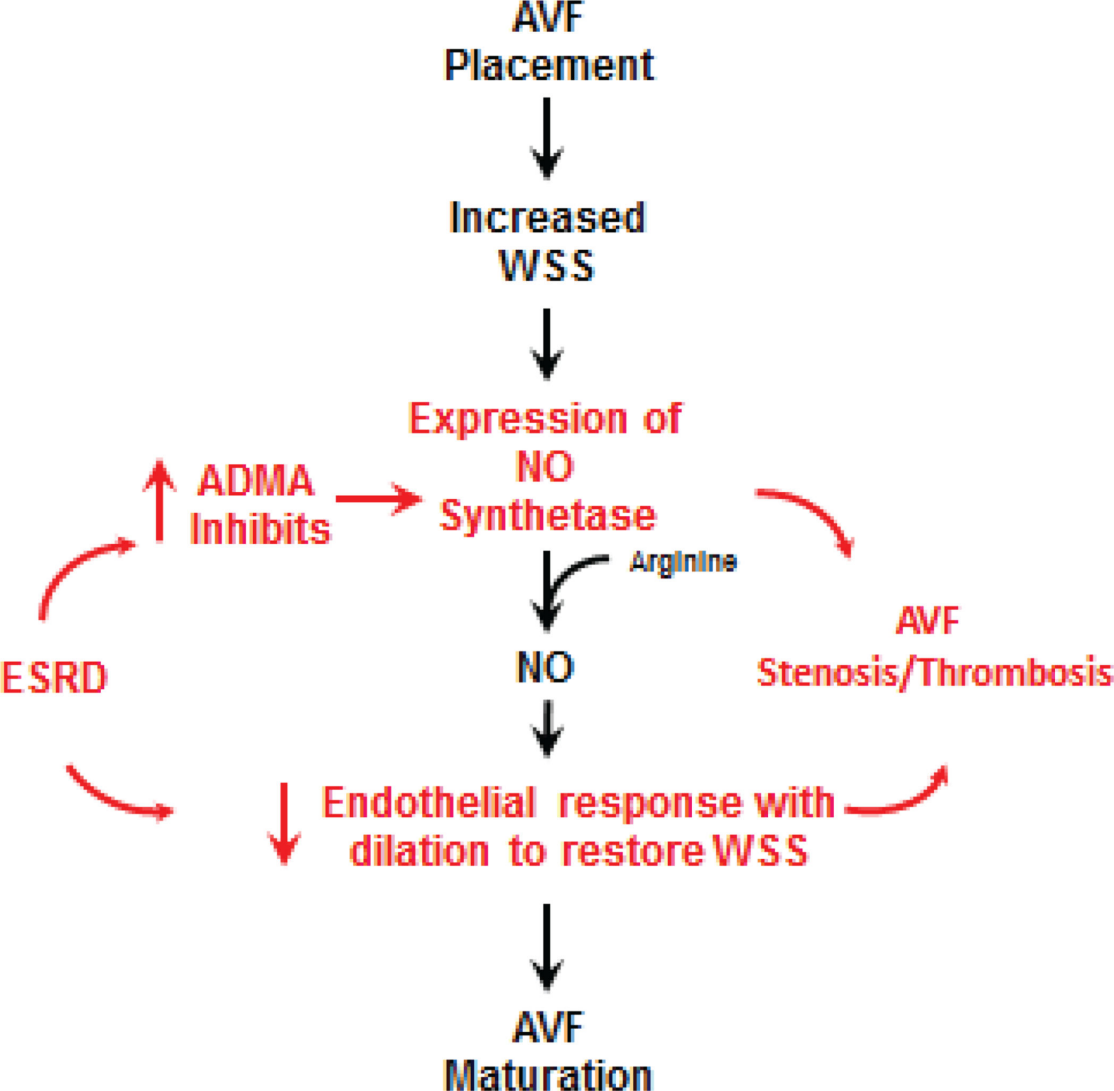
Schematic of hypothesis ADMA contributes to AVF stenosis and thrombosis ADMA: Asymmetric Dimethylarginine; AVF: Arteriovenous Fistula; ESRD: End-Stage Renal Disease; NO: Nitric Oxide; WSS: Wall Shear Stress.

Our purpose was to test the hypothesis that increased ADMA, which occurs in renal failure patients, with resultant decreased endothelial cell response to NO would predict adverse events of stenosis and thrombosis in patients with AVF access (Figure 1). The primary aim was to describe the associations of ADMA with fistula survival and venography findings in a cohort of ESRD patients with newly constructed AVF access. A secondary aim was to determine if other demographic factors influenced the outcome of the AVF.

## Methods

This project was part of a prospective, observational cohort study evaluating fistula outcomes that was conducted at a large, single-center university-based medical center from October 7, 2011 to September 5, 2016. Subjects were recruited sequentially when it was determined that they would have a primary brachiocephalic fistula placed and agreed to be followed by the study schema giving written consent approved by the IRB at the University of Chicago (IRB 11-0269). The data presented in the current study was a subset of a larger study to determine if low WSS in the cephalic arch contributed to cephalic arch stenosis. The study was sufficiently powered for the statistical analysis of the primary outcome of cephalic arch stenosis, but was not prospectively powered for the secondary outcome of stenosis/thrombosis presented in this paper. Patients were included in the study if a primary BCF was placed for dialysis vascular access, with no previous access in the ipsilateral arm and if a preoperative blood sample for ADMA was obtained. Patients were excluded if a blood sample at for ADMA could not be obtained at time of AVF creation.

### Patient characteristics

Patient demographics and clinical parameters of interest included: gender, ethnicity, age at time of surgery, dialysis days prior to access placement, and history of diabetes, hypertension, coronary artery disease, or peripheral vascular disease.

### ADMA

ADMA concentration in plasma samples was measured as described previously by Lazich et al. [21,22]. Briefly plasma was separated from 10 ml blood samples drawn preoperatively, on the day of fistula placement. 200 µL of plasma was mixed with 2.5 µL of homoarginine (internal standard) and larger proteins were precipitated using acetonitrile. This sample was then derivatized using a 2% ninhydrin ethanolic solution. After the color development step, the sample was loaded into an Agilent LC system. Separation was achieved by isocratically delivering the sample mixed with mobile phase “A” (13 mM Ammonium bicarbonate with 10% (v/v) tetrahydrofuran) through a 4.6 mm × 15 cm Agilent Extend C18 column, at 1.3 mL/min. Fluorescence signal at 497 nm (emission) from the sample was recorded by exciting the sample through the flow cell at 390 nm (excitation). The peak from homoarginine was recorded at 5.5 minutes post injection and that from ADMA was recorded at 11.5 minutes. The peak area of the internal standard was used to calculate the concentration of ADMA in samples.

### Venogram

**Figure 2:**
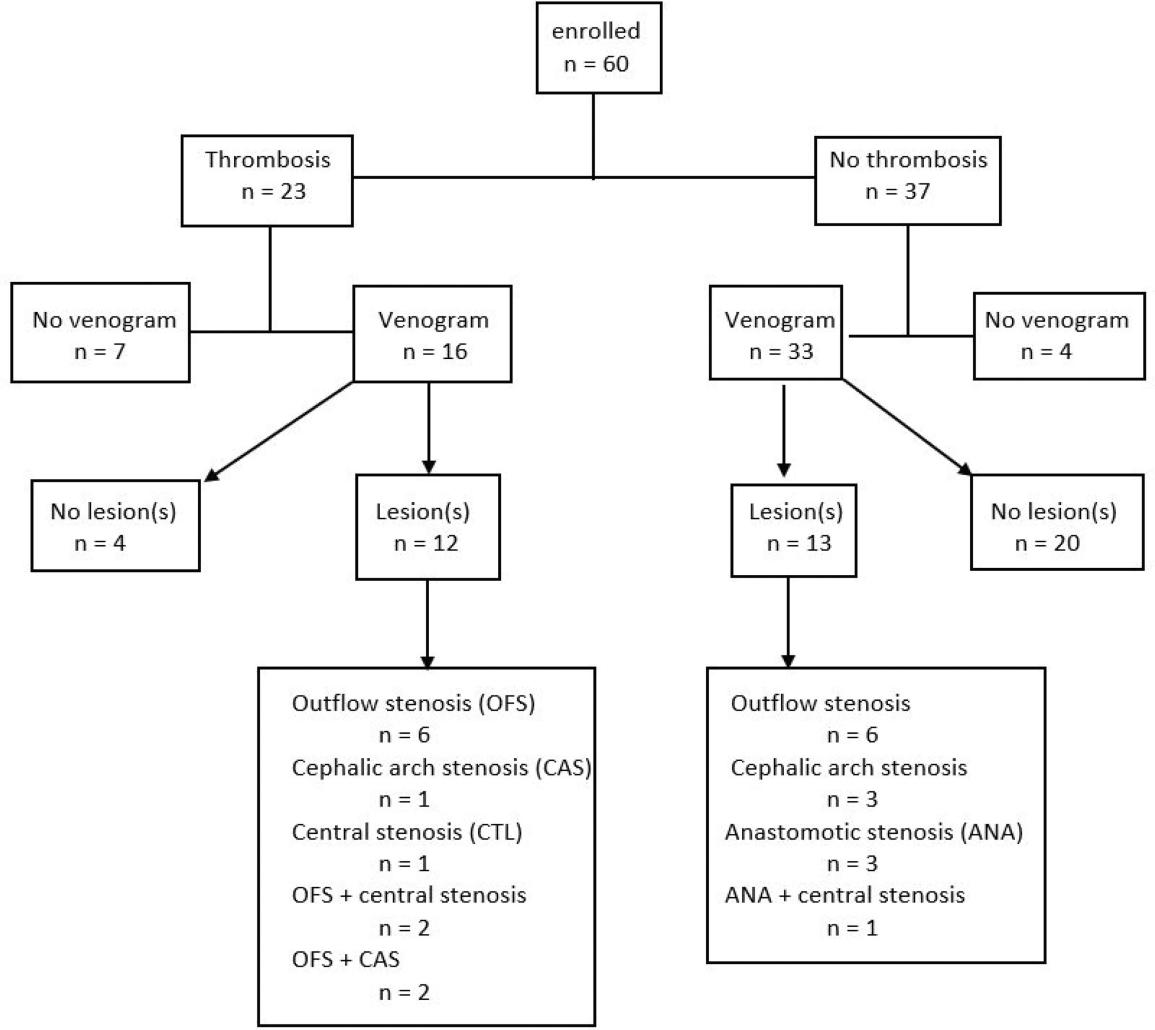
Flow diagram of Cohort OFS: Outflow Stenosis; CAS: Cephalic Arch Stenosis; CTL: Central Stenosis; ANA = Anastomotic Stenosis.

Venography was performed at approximate time of maturation, yearly for up to three years, or as clinically indicated. The time of maturation was defined as the time when the AVF was successfully cannulated with 2 needles for three sequential treatments. The time to maturation varied from 3–6 months. The fistula was punctured using a micropuncture system near the anastomosis with contrast injection towards the venous limb. The needle was exchanged for a 5 French dilator, and digital subtraction venography encompassing the outflow from puncture site to the right heart was performed. Venous stenosis was defined as narrowing in the vein as compared with the diameter from the upstream vein and angioplasty was performed if the stenosis was greater than 50% and the patient had a clinical indication (such as poor clearance, high venous pressure or prolonged bleeding).The stenosis was recorded as defined by anatomic location as follows: venous outflow stenosis if it occurred past the anastomosis but distal to the central vessels; as cephalic arch stenosis if it occurred in the bend of the cephalic vein proximal to the connection of the axillary vein; or as central vein stenosis when it occurred distal to the axillary vein (Table 1 and Figure 2).

### Operative technique for BCF creation

The patient had a fistula created as follows: the cephalic or the median cubital vein was dissected free using a combination of electrocaudary, blunt, and sharp dissection. The brachial artery was isolated in a similar fashion. The proximal end of the vein was controlled. The distal end of the vein was ligated, and the vein was divided. The artery was controlled both at the proximal and distal aspect. A longitudinal arteriotomy was made which was irrigated with heparinized saline, and an end vein to side artery anastomosis was completed. Vascular control was released. There was a Doppler signal only in the outflow vein and a palpable pulse in the artery distal to the anastomosis. The wound was irrigated and the incision was closed.

### Outcomes

For each patient, the date of access surgery was taken as the baseline time zero, and clinical course was followed until time of access thrombosis or September 5, 2016, whichever came first. The primary outcomes were time to access thromboses and occurrence of venous stenosis.

### Statistical analysis

Means and medians were used to summarize normally and non-normally distributed continuous variables. Student’s t test, Wilcoxon rank sum tests, chi-squared tests, and Fisher’s exact test were used to compare baseline values, as appropriate. A significance level of 0.05 corresponded to a confidence intervals of 95%. Competing risks regression was performed to evaluate associations with time to thrombosis. A cumulative incidence plot was used to compare event-specific thrombosis outcomes between individuals with above-median ADMA levels to those with below-median ADMA levels. Adjustment variables were selected *a priori*, based on their relevance to the outcomes of fistula failure, and included age, sex, body mass index, hemodialysis vintage, diabetes, coronary artery disease, and peripheral vascular disease. Hypertension was present in nearly all patients, and not adjusted for. Venous diameter was measured only infrequently in patients with thrombosis, and therefore omitted from the multivariable model. Loss to follow up was censored; death and kidney transplantation were treated as competing events. Follow up continued until thrombosis, a competing event, or September 5, 2016, whichever came first.

As detection of stenosis was dependent upon elective scheduling of venography, time to event analyses were not performed for these outcomes, in the subset of patients who had venography performed. Logistic regression was used to evaluate the associations of ADMA with the composite outcome of any venous stenosis, and models were adjusted for age, sex, body mass index (BMI), months of hemodialysis prior to surgery, diabetes, coronary artery disease, and peripheral vascular disease, and venous diameter at venography. All analyses were conducted using SAS, University Edition (SAS Institute Inc., Cary, NC).

## Results

105 patients were initially evaluated for study entry. Of these potential participants, 45 were excluded. The major reasons for exclusion were: blood sample not available for ADMA (n=12), death prior to access placement or a maturation venogram (n=14), withdrawal (n=5), loss to follow up (n=4), and change of modality (n=10). Table 1 summarizes the study population, which consisted of 60 patients who had a primary brachiocephalic AVF placed and had a blood sample for ADMA at the time of surgery. The average age of the population was 57.5 years and 50% were female. Median follow-up was 221 days (interquartile range (IQR)=92, 365 days). The median value for ADMA was 3.1 µmol/L with a range of 0.9–13.0 µmol/L. Thromboses were observed in 19 individuals. 4 subjects had a primary thrombosis resulting in failure diagnosed within 2 weeks after AVF was placed, these AVF were never used for dialysis. 8 subjects had primary thrombosis resulting in failure diagnosed by three months, these AVF were considered failed and were not used for hemodialysis. 7 subjects had a thrombosis after AVF placement with successful declot and the AVF was still used for dialysis.

Among patients who did not have thrombotic events, one patient was lost to follow up, seven patients died, and four patients had kidney transplantation. The proportion of diabetics was significantly higher in patients who sustained a thrombotic event. Among the 49 patients who underwent venography either by protocol (n=36) or due to thrombosis (n=13), 25 had venous stenosis detected and 24 did not (Figure 2). Anticoagulants or other platelet inhibitors did not predict thrombosis (Table 1).

**Figure 3:**
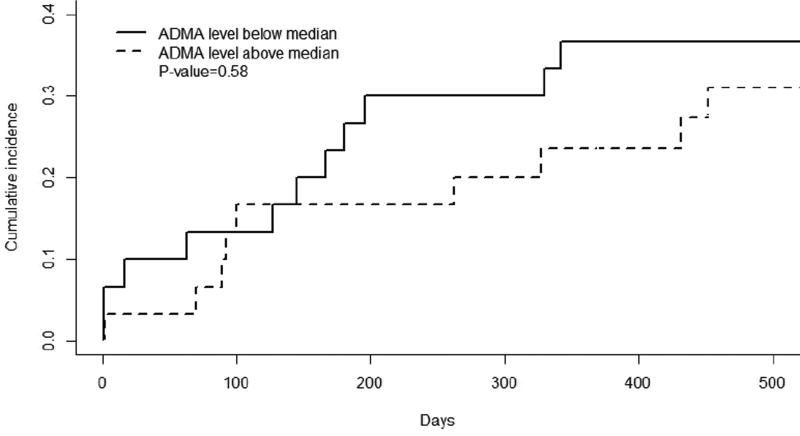
Kaplan-Meier Survival Plot, Time to Thrombosis, by ADMA level Cumulative incidence plot showing no significant differences in time to thrombosis, stratified by below-median versus above-median levels of asymmetric dimethylarginine (ADMA)

Kaplan-Meier curves for thrombosis-free survival in the groups with above-median and below-median ADMA levels were nearly identical (Figure 3). When ADMA levels were separated into quartiles and correlated with outcomes either stenosis, any stenosis or thrombosis, thrombosis or non-maturation there was no significant difference (p>0.05). The competing risks regression model confirmed the significant effect of diabetes on the hazard of thrombosis, but revealed no associations of ADMA level with thrombosis in either the univariate or the adjusted models (Table 2). Similarly, the logistic model did not reveal a significant association between ADMA level and early venous stenosis (Table 3). When the events of stenosis and thrombosis were combined there was no association with the level of ADMA obtained at time of AVF creation (data not shown). Similarly, the logistic model did not reveal a significant association between ADMA level and early venous stenosis (Odds Ratio (OR)=1.07, 95% confidence interval 0.84, 1.34, *P*=0.60).

## Discussion

Preoperative ADMA levels were not associated with early stenosis or a thrombotic event at one-year in patients on hemodialysis with BCF access. We measured early events in AVF maturation, thrombosis and venous stenosis. Prior studies have looked at the association of ADMA with symptomatic restenosis after percutaneous angioplasty of AVF. The cohort included in previous studies was receiving hemodialysis for at least 6 months. We chose to study patients with primary BCF access and evaluated initial events within the first year of access use.

Reference values for ADMA in CKD and ESRD range between 3–15 µmol/L [18]. Our results for patients with ESRD are consistent with published reference range, but differ from those published in previous investigation by Wu et al, whose median value for ADMA by ELISA assay was 0.91 µmol/L [19]. This is far below what would be expected in ESRD [23]. The current analysis utilized HPLC, which is the gold standard for ADMA measurement. Our results using HPLC show much higher plasma ADMA concentrations in ESRD patients compared to those from the study published by Wu et al. [19]. This can be explained by the higher sensitivity of measurements using HPLC. Historically, ELISA was used as the most common technique for ADMA detection in plasma. The sensitivities of most ELISA kits available for these measurements are in the range of 0.05 µmol/L. The HPLC method is accurate on the order of nmol/L, leading to a higher resolution over smaller sample volumes. HPLC measurement is further improved by the use of a higher precision detector than the normal fluorescence detectors in 96-well plate readers. Additionally, the HPLC method utilizes standard chemical derivatization with a relatively stable dye (ninhydrin) at working conditions, unlike ELISA’s reliance upon the viability of antibodies, and their specificity to ADMA. The derivatization process also eliminates larger proteins present in the serum/plasma and the pore size on the column ensures highly sensitive separation of proteins, even with very small differences in molecular weight. This further ensures, methodologically, accurate collection of fluorescence signal from the sample of interest. The correlation between commercial ELISA kit for the quantitative analysis of ADMA and HPLC is poor [24,25], which may in part explain the discordant results. In summary, HPLC offers better sensitivity and specificity when determining ADMA measurements.

The limitations of this study include that it was conducted in a single center, some subjects had missing data, and use of a single ADMA measurement. The time resolution at which the current data was collected, is limiting. Whereas the process of AVF maturation takes several weeks, the current data was collected at the time of fistula placement (pre-operative). A logical extension to the current study would be to assess ADMA concentration on a daily or weekly basis after AVF placement. It would also be of interest to follow the ADMA measurements for the lifespan of the BCF. The total number of patients and events provides low power for negative findings. An additional limitation is that the population studied was an American dialysis population of African American patients and may not be generalized to other populations from other countries or diverse ethnic origin. We cannot rule out the possibility that small effects may have been detected, had more patients been evaluated. In addition, we were unable to evaluate other products of arginine metabolism, such as the arginine/ADMA ratio or symmetric dimethyarginine (SDMA), which might have provided further insights. While SDMA doesn’t directly inhibit NO, it may compete with membrane transporters of the endothelial cell altering the level of NO [26].

The availability of predictive biomarkers could play an important role for physicians to decide the best route for vascular access for hemodialysis for ESRD patients. Albeit being the most common technique to gain vascular access, AVFs have a high failure rate. This can be mitigated with the use of an alternative method of access or intervention to aid the maturation of the fistula. However, research into such biomarkers is confounding and laborious. The results of the current study are a step in the right direction towards establishing an understanding of the contributing factors for AVF failure.

In summary, the vascular endothelium and ability to produce adequate NO is central to adequate AVF maturation. The elasticity (stiffness) of vessels does affect AVF maturation [27]. Although serum ADMA, an inhibitor of NO, causes vessel stiffness, we were unable to show an association with early maturation failure events. As inflammation is an important determinant of endogenous nitric oxide mediated by high ADMA levels it would be of interest to follow prospective cohorts of patients with ESRD for trends in CRP and ADMA. The biologic effects of ADMA contributing to venous stenosis in patients with AVF are the subject of future research investigation, not just preoperative measures but also post intervention, as ADMA levels or persistence of high ADMA levels could have important clinical implications. Future studies should continue to investigate biomarkers for physiological pathways that lead to inadequate production of NO and dysfunctional endothelial cell function [28].

## Figures and Tables

**Table 1:** Characteristics of patients who did and did not have thrombosis

	All (n=60)	Thrombosis (n=19)	No thrombosis (n=41)	P-value
Age, mean (std)	57.5 (15.2)	59.3 (12.7)	56.4 (16.6)	0.5
Female sex, n (%)	30 (50.0)	9 (39.1)	21 (56.8)	0.2
ADMA µmol/L, median (IQR)	3.1 (2.2, 4.7)	3.0 (1.8, 4.4)	3.6 (2.4, 5.0)	0.4
Months on HD, median (IQR)	3.4 (1.9, 8.7)	3.0 (1.4, 8.7)	3.4 (2.0, 7.8)	0.4
Venous diameter mm, mean (std)	5.4 (2.1)	4.7 (1.7)	5.6 (2.2)	0.2
Body mass index, mean (std)	30.6 (8.1)	31.6 (9.4)	29.9 (7.2)	0.4
n, vein diameter measured	42	10	32	
Clinical Parameters, n (%)				
Hypertension	57 (95.0)	22 (95.7)	35 (94.6)	0.9
Diabetes	35 (58.3)	17 (73.9)	18 (48.7)	0.05
Aspirin use	35 (58.3)	12 (52.2)	23 (62.2)	0.4
Warfarin use	8 (13.3)	3 (13.0)	5 (13.5)	0.9
Coronary artery disease	2 (3.3)	0 (0)	2 (5.4)	0.3
Clopidergrel use	22 (36.7)	8 (34.8)	14 (37.8)	0.8
Peripheral vascular disease	12 (20.0)	6 (26.1)	6 (16.2)	0.4
n, venography performed	49	16	33	
Radiographic findings, n (%)				0.01
No stenoses	24 (49.0)	4 (25.0)	20 (60.6)	
Outflow vein stenosis	12 (24.5)	6 (37.4)	6 (18.2)	
Mixed stenoses	5 (10.2)	4 (25.0)	1 (3.0)	
Cephalic arch stenosis	4 (8.2)	1 (6.3)	3 (9.1)	
Anastomotic stenosis	1 (6.1)	0	3 (9.1)	
Central stenosis	1 (2.0)	1 (6.3)	0	

Abbreviations: ADMA: Asymmetrical Dimethyl Arginine; IQR: Interquartile Range; mm: millimeter

**Table 2:** Univariate and multivariable hazard ratios (HR) for associations with time to thrombosis

	Univariate			Multivariable*		
	HR	95% CI	P	HR	95% CI	P
Serum ADMA, µmol/L	0.92	0.76, 1.10	0.3	0.85	0.65, 1.11	0.2
Age, per 10 years	1.07	0.86, 1.33	0.5	1.00	0.97, 1.04	1.0
Female sex	0.67	0.29, 1.55	0.3	0.35	0.07, 1.68	0.2
Months on hemodialysis	1.00	0.97, 1.03	0.9	1.02	0.99, 1.06	0.2
Body mass index	1.01	0.96, 1.07	0.6	1.01	0.92, 1.13	0.8
Venous diameter, mm	0.82	0.60, 1.12	0.2			
Hypertension	1.04	0.10, 10.5	1.0			
Diabetes	2.55	1.01, 6.43	0.05	2.68	0.85. 8.40	0.09
Coronary artery disease	0.86	0.37, 2.00	0.7	0.61	0.09, 3.90	0.6
Peripheral vascular disease	1.48	0.61, 3.60	0.4	2.47	0.33, 18.6	0.4
Aspirin use	0.71	0.32, 1.59	0.4	0.43	0.11, 1.68	0.2

* Variables omitted from multivariate model: hypertension present in >90% of both groups; venous diameter measured in only 8 of 19 patients with thrombosis.

ADMA: Asymmetric Dimethyl Arginine; mm: millimeter; HR: Hazard ratio; CI: Confidence Interval

**Table 3:** Univariate and multivariable odd ratios (OR) for associations with any venous stenosis

	Univariate			Multivariable		
	OR	95% CI	P	OR	95% CI	P
Serum ADMA, µmol/L	1.07	0.84, 1.34	0.6	1.13	0.84, 1.50	0.4
Age, per 10 years	0.87	0.61, 1.23	0.4	0.81	0.52, 1.26	0.3
Female sex	2.10	0.67, 6.56	0.2	2.83	0.64, 12.5	0.2
Months on hemodialysis	1.00	0.96, 1.05	0.9	1.01	0.96, 1.07	0.7
Body mass index	0.95	0.88, 1.03	0.2	0.95	0.85, 1.06	0.4
Venous diameter, mm	1.16	0.86, 1.56	0.4	1.23	0.84, 1.79	0.3
Diabetes	1.54	0.49, 4.81	0.5	3.46	0.71, 16.9	0.1
Coronary artery disease	0.50	0.15, 1.70	0.3	0.50	0.09, 2.84	0.4
Peripheral vascular disease	1.05	0.23, 4.78	0.9	0.60	0.08, 4.66	0.6
Aspirin use	1.10	0.36, 3.41	0.9	1.06	0.22, 5.06	0.9

OR: Odds ratio; CI: Confidence Interval; ADMA: Asymmetric Dimethyl Arginine
